# Ecology, adaptation, and function of methane‐sulfidic spring water biofilm microorganisms, including a strain of anaerobic fungus *Mucor hiemalis*

**DOI:** 10.1002/mbo3.483

**Published:** 2017-05-24

**Authors:** Enamul Hoque, Johannes Fritscher

**Affiliations:** ^1^ Helmholtz Zentrum München GmbH – German Research Center for Environmental Health Institute of Groundwater Ecology Neuherberg Germany

**Keywords:** Anaerobes, *Archaea*, biofilm, microbial ecology

## Abstract

Ecological aspects, adaptation, and some functions of a special biofilm and its unique key anaerobic fungus *Mucor hiemalis* strain EH11 isolated from a pristine spring (Künzing, Bavaria, Germany) are described. The spring's pure nature is characterized by, for example, bubbling methane, marine‐salinity, mild hydrothermal (~19.1°C), sulfidic, and reductive‐anoxic (E_h_: −241 to −253 mV, O_2_: ≤ 0.1 mg/L) conditions. It is geoecologically located at the border zone between Bavarian Forest (crystalline rocky mountains) and the moor‐like Danube River valley, where geological displacements bring the spring's water from the deeper layers of former marine sources up to the surface. In the spring's outflow, a special biofilm with selective microorganisms consisting of archaea, bacteria, protozoa (ciliate), and fungus was found. Typical sulfidic‐spring bryophyta and macrozoobenthos were missing, but many halo‐ and anaerotolerant diatoms and ciliate *Vorticella microstoma* beside EH11 were identified. Phase contrast and scanning electron microscopy revealed the existence of a stabilizing matrix in the biofilm formed by the sessile fungal hyphae and the exopolysaccharide substance (EPS) structures, which harbors other microorganisms. In response to ecological adaptation pressure caused by methane bubbles, EH11 developed an atypical spring‐like hyphal morphology, similar to the spiral stalk of ciliate *V. microstoma*, to rise up with methane bubbles. For the first time, it was also demonstrated that under strict anaerobic conditions EH11 changes its asexual reproduction process by forming pseudosporangia via hyphal cell divisions as well as switching its metabolism to chemoautotrophic bacteria‐like anaerobic life using acetate as an e‐donor and ferrihydrite as an e‐acceptor, all without fermentation. EH11 can be suggested to be useful for the microbial community in the Künzing biofilm not only due to its physical stabilization of the biofilm's matrix but also due to its ecological functions in element recycling as well as a remover of toxic metals.

## 1 INTRODUCTION

In contrast to fresh water ecosystems, sulfidic springs at various hydrogeological interfaces present unique microbial ecological habitats because of special physical and chemical conditions, for example, reducing/oxidizing, sulfidic, hydrothermal/cold, anoxic/hypoxic, marine‐salinity, and presence of methane and heavy metals. They are mainly fed by deep ground waters of different qualities and are mixed with younger water in various proportions; some are influenced by anthropogenic activities (Heinrichs, Hoque, Wolf, & Stichler, 2000; Hoque & Fritscher, 2016; Hoque, Pflugmacher, Fritscher, & Wolf, 2007). Selective biofilms and microbial community members of various evolutionary origins grow there, some of which are biotechnologically important (Hoque & Fritscher, 2016; Hoque et al., 2007). The sulfidic Künzing spring is an exceptional example due to bubbling methane of deep origin (Carle 1975). Aquatic fungi of sulfidic springs are filamentous microorganisms embedded in the EPS structures; sometimes mixed with microalgae that adapts itself with unique detoxification functions (Hoque & Fritscher, 2016; Hoque et al., 2007). In the case of Künzing spring, due to flushing away of dissolved oxygen by continuous methane evolution, we can expect a special anaerobic microbial community resistant to bubbling methane and low oxygen levels. In other sulfidic springs, the biofilm matrices were mainly stabilized by aquatic fungi that offer physical support for other microorganisms essential for their existence and dispersal in this special aquatic ecosystem.

Although fungi can be facultative anaerobic (Deacon, 1984; Kurakov, Lavrent′ev, Nechitailo, Golyshin, & Zvyagintsev, 2008), some of them from the orders *Hypocreales*,* Eurotiales*,* Mortierellales,* and the phylum *Zygomycota* can live for a month under fermentative anaerobiosis (Kurakov et al., 2008). Strictly anaerobic rumen fungi have been reported (Flint, 1997). Research on anaerobic fungi deserves our attention because they are useful for the bioavailability and nutrient cycling of organic carbon from complex polysaccharides of plant cell walls (Flint, 1997; Gerbi et al., 1996). In this way, they play a significant role in feeding process of ruminants (Chaudhry, 2000; Gordon & Phillips, 1998; Lee, Shin, Kim, Ha, & Han, 1999). They are also useful for many biotechnological applications as they produce noble overexpressed enzymes including diverse cellulase, hemicellulase, chitinase, endoglucanase, and xylanase (Bhat & Bhat, 1997; Flint, 1997; Lee et al., 1999; Yanke, Selinger, Lynn, & Cheng, 1996). They interact with the H_2_‐utilizing acetogens beside methanogens or sulfate‐reducing bacteria in the microbial community of anaerobic ecosystems (Morvan, Rieu‐Lesme, Fonty, & Gouet, 1996). They are apparently significantly responsible for the DOC (Dissolved Organic Carbon) release from complex poorly degradable ligno‐cellulosic materials and for carbon cycling in the environment (Flint, 1997). Fungal release of DOC from complex organic materials may have major influences on the microbial community structures that utilize DOC as electron donors, especially in anaerobic environments.

In contrast to anaerobic rumen fungi, we know little about aquatic anaerobic fungi. It was previously reported that some yeasts and filamentous fungi like *Fusarium oxysporum*,* Mucor hiemalis,* and *Aspergillus fumigatus* were able to grow in absence of oxygen by fermenting sugars (Deacon, 1984). There were also several previous reports about some aquatic fungal strains with the ability to live under anaerobiosis, for example, under oxygen‐limited conditions of sewage sludges, polluted waters, organic‐enriched soils (Tabak & Cooke, 1968), and submerged rice fields (Tonouchi, 2009; Wada, 1976), apparently via fermentation of substrates (Tonouchi, 2009). Aeroaquatic hyphomycetes showed variations in tolerance to anoxic conditions, some of them *Helicodendron triglitziense, H. conglomeratum,* and *H. giganteum* as well as *Saprolegnialesm* species with oospores survived up to 3 months under anaerobic conditions (Field & Webster, 1983). Using various sterilized wood probes in lysimeters, we could show differential affinity of some facultative anaerobic fungi to these wood probes in subsurface at depths ≤5 m (Hoque & Klotz, 2002). Similarly, Krauss et al. (2003) placed sterilized *Alnus glutinosa* leaf disks in wells and showed the occurrence of aquatic hyphomycetes in subterranean environment, especially in polluted groundwater habitats. It can be assumed that the aquatic hyphomycetes, as well as other facultative anaerobic fungi, play an important role in C cycling from decomposition of litter and woody materials in subsurface waters.

In contrast to subsurface waters and underground soil influenced by rain water, sulfidic springs are mainly fed by deep anaerobic groundwater (Heinrichs et al., 2000) and, as such, are more suitable for the search of fungi living strictly anaerobically. Until now not a single report on the occurrence of strictly anaerobic fungi in groundwater is available that can show their ability to live in anoxic groundwater ecosystems without fermenting sugar. Occurrence of aerobic or facultative anaerobic fungi in subsurface groundwater–sediment systems of varying depths is known (Hoque & Klotz, 2002; Hoque et al., 2007). One bottle neck of research on anaerobic fungi in groundwater could be the lack of suitable methodologies for cultivation, and morphological and functional analyses of aquatic anaerobic fungi in liquid cultures.

After our thorough search of suitable anaerobic biofilms among sulfidic spring water biofilms, Künzing spring's biofilm offered a good opportunity to look for anaerobic fungi and to develop research methodologies. In order to withstand the pressure and anaerobiosis created by bubbling methane and reducing conditions, the biofilm growing there was adapted to anoxic conditions and stabilized by some anaerobic microorganisms like anaerobic fungi that can build 3D physical structures bound to the rocks. Therefore, the major objectives of our studies are to (1) establish methodologies for a systematic search of strictly anaerobic fungi in groundwater–sediment ecosystems based on both cultivation‐dependent and cultivation‐independent molecular biological approaches, (2) to unlock the ecological aspects of aquatic anaerobic fungi, (3) to demonstrate the morphology and strictly anaerobic life of a new *Mucor hiemalis* strain EH11 isolated from nearly anoxic methane‐sulfidic spring water, (4) to show the utilization of acetate as an e‐donor and ferrihydrite as an electron acceptor with AQDS as an electron shuttle under strictly anoxic conditions, (5) to present the ecological importance and functions of EH11 in removing various toxic metal ions simultaneously, as well as (6) to explore the role of aquatic anaerobic fungi in helping establishment of diverse life at the special deep sea–like terrestrial interface.

## 2 MATERIALS AND METHODS

### 2.1 Locality and hydrogeochemistry of Künzing spring

The methane‐sulfidic subthermal salt spring of Künzing is located at latitude 48°39′56.922084′′ N and longitude 13°5′42.135324′′ E about 306 m over NN in Bavaria, Germany. Some characteristic parameters like oxygen, conductivity, redox potential, and sulfide concentrations were measured at the spring site. Sulfide was measured by Microquant kit (Merck, Darmstadt, Germany). Temperature and pH were recorded using a portable pH meter (E50‐pH electrode) equipped with a thermometer (Model 196, WTW, Weilheim, Germany). Oxygen concentration was measured using an oxygen measuring instrument (Model Oximeter OXI 197‐S, WTW) equipped with an oxygen‐specific sensory cell (OX 325, WTW, Germany). The electrical conductivity and the redox potential were determined by using a Konduktometer LF 196 equipped with the TetraCon 96‐1.5 electrode (Model 196, WTW) and the Pt 4805 electrode (Model 196, WTW), respectively.

The spring water samples were filtered through 2‐μm filter and stored in a fridge (4°C) awaiting subsequent analyses. Cations (e.g., Na^+^; K^+^, NH_4_^+^, Mg^2+^, Ca^2+^, Mn^2+^) and anions (e.g., Cl^−^, Br^−^, NO^3−^, SO_4_^2−^, HCO_3_^−^) of water were determined by ion chromatography (IC) coupled with an electrochemical detector (Dionex DX 100, Dionex Softron, Germering, Germany) as described (Hoque et al., 2007). Dissolved organic carbon (DOC) concentration was measured following NPOC method as reported (Hoque et al., 2007). The ionic metals (Al, As, Cd, Co, Cr, Cu, Hg, Li, Ni, Pb, Sr, and Zn) after complexation with EDTA were analyzed and quantified by using Dionex 500 system by using inductively coupled plasma‐optical emission spectrometry (ICP‐OES) for the quantitative and qualitative detection of elements (Hoque et al., 2007). Water age was determined by tritium‐ (Quantulus 1220, Perkin Elmer, Rodgau, Germany) and ^14^C‐dating techniques (Heinrichs et al., 2000).

### 2.2 Biofilm collection and enrichment culture

Fresh biofilms (2–5 g) from submerged rocks in Künzing spring were collected in sterile falcon tubes and immediately placed on dry ice. The biofilms were transported on dry ice to the laboratory, and immediately centrifuged at 4,000*g* (5 min) to concentrate as biomass pellets. They were either subjected to DNA extraction (see below) or used as inoculants on sterile malt extract (30 g L^−1^)‐agar (15 g L^−1^) solid media (Arjmand & Sandermann, 1985) supplemented with 100‐ppm streptomycin sulfate. Repeated reinoculations and cultivations led to pure fungal cultures, which were observed under stereo microscopy (MZ16, Leica, Wetzlar, Germany) and phase contrast microscopy (Axiolab, Zeiss, Oberkochen, Germany) after a modified safranin staining (Hoque et al., 2007). The sterilized biofilms of Künzing spring and the biofilms of springs Wildbad Kreuth, Bad Abbach, and Pilzweg devoid of *Mucor* sp. served as negative controls for comparison.

### 2.3 Biodiversity and identification of fungus in biofilms

Identification of fungus by using morphological, zygote formation (crossing experiment with corresponding minus‐strand), and fluorescence in situ hybridization (FISH) analysis data were tested for various biofilms of different springs and carried out as described (Hoque et al., 2007) as well as performed by using electron microscopy as described (Hoque & Fritscher, 2016). DNA from Künzing biofilm was prepared and purity checked according to our protocol previously published (Hoque et al., 2007). Primer pairs selected from literature were as follows: (1) EF4(f)/FF390(b) (Smit, Leeflang, Glandorf, van Elsaas, & Wernars, 1999; Vainio & Hantula, 2000), (2) ITS1F(f)/ITS4(b) (Gardes & Bruns, 1993; White, Bruns, Lee, & Taylor, 1990), and (3) EF3RCNL(f)/ITS4(b) (Lord, Kaplan, Shank, Kitts, & Elrod, 2002; White et al., 1990). After optimization we found ITS1F(f)/ITS4(b) as the best primer pair for the amplification of fungal 18S rRNA gene sequence (Hoque et al., 2007) and of ITS1‐5.8S‐ITS2 rRNA regions for cloning and sequence analysis (see below).

The FISH analysis for in situ investigations of biofilms was performed at least in duplicates and described in detail (Hoque et al., 2007). The FISH protocol for the detection of aquatic *Mucor hiemalis* was tested and verified using biofilms of various sulfidic springs (Hoque et al., 2007). For FISH analysis, formaldehyde‐fixed samples (*Archaea*,* Bacteria*,* Fungi*, biofilms) were suspended in wells of carrier slide (Paul Marienfeld, Lauda‐Königshofen, Germany). They were fixed to glass wells by passing quickly through a bunsen burner and then dehydrated in ethanol gradient (50%, 80%, and 100%). After dehydration, they were air dried and in situ hybridized with known fluorescently labeled oligonucleotide probes targeting *Mucor hiemalis* (MH1; Hoque et al., 2007), *Archaea* (ARCH915 for all *Archaea*, Stahl & Amann, 1991; EURY498 for *Euryarchaeota*, CREN499 for *Crenarchaeota*, Burggraf et al., 1994), *Proteobacteria* (ALF1b for α*‐Proteobactera*; BET42a for β*‐Proteobacteria*; GAM42a for γ*‐Proteobacteria*, Manz, Amann, Ludwig, Wagner, & Schleifer, 1992; DELTA495a,b,c for δ*‐Proteobacteria*, Loy et al., 2002), *Cytofaga* and *Flavobacteria* (CF; Wagner et al., 1994), high G + C *Bacteria* (HGC, Roller, Wagner, Amann, Ludwig, & Schleifer, 1994), *Eubacteria* (EUB338, Amann et al., 1990), and all microorganisms (UNIV1392, Stahl, Flesher, Mansfield, & Montgomery, 1988) as described (Hoque et al., 2007). After embedding in citifluor, fluorescent images of labeled microorganisms were taken either at a laser‐scanning confocal fluorescence microscope (LSM 510, Zeiss) equipped with a photomultiplier imaging system (Zeiss) or at an Axiolab or Axiovert fluorescence microscope equipped with a Leica ocular photography adapter for digital cameras (Zeiss).

### 2.4 Fungal cultivation

The fungal strains of sulfidic springs and ground water sediments (mainly water‐saturated bore hole core section from 10.35 m depth, Kellermann et al., 2012) were cultivated in liquid culture medium (see below) and on malt extract agar solid medium supplemented with streptomycin sulfate (100 mg/L) under anaerobic and aerobic/anaerobic N_2_‐flushed conditions, respectively. Temperature range was optimized for optimum growth. A stereo microscope was used for morphological investigation of the fungi and a digital camera was used for documentation.

### 2.5 Anaerobic liquid cultures of EH11

Various liquid culture media of bacteria (Widdel, 1980) and fungi (Caldwell & Bryant, 1966; Kirk, Schultz, Connors, Lorenz, & Zeikus, 1978) for anaerobic cultivation of aquatic fungi were repeatedly tested. The growth parameters (e.g., medium composition, electron acceptor, electron donor) for the cultivation and assays of aquatic anaerobic fungi from groundwater–sediment samples (see above) were varied, modified, and optimized each time in triplicate (*n* = 3). A typical optimized composition of final anoxic liquid medium developed for strictly selective growth of anaerobic aquatic fungi at optimum temperature was as follows: sodium hydrogen carbonate (30 mM) plus sodium carbonate (4.4 mmol L^−1^) (pH 6.5), acetate (50 mmol L^−1^, electron donor), ferrihydrite (2 mmol L^−1^, electron acceptor), AQDS (9,10‐anthraquinone‐2,6‐disulfonate, 2 mmol L^−1^), ammonium hydrochloride (24 mmol L^−1^), Tween 80 (0.1%), macroelement solution (Kirk et al., 1978; 2.8%), trace element solution (Kirk et al., 1978; 0.26%), streptomycin sulfate (100 mg L^−1^), lysozyme (40 mg L^−1^) plus EDTA (30 mg L^−1^), Penicillin G (1 K units L^−1^), vitamin solution (Kirk et al., 1978; 200 μl L^−1^), sodium sulfide (0.5 mmol L^−1^), cysteine hydrochloride (0.03%), resazurin (0.0001%), prewashed jute fibers (1 cm long, 20 pieces/L), saturated with N_2_:CO_2_ (v/v) = 80:20, and sterilized under anaerobic conditions.

### 2.6 Light and electron microscopy as well as elemental analysis

The biofilms were observed under in situ intact conditions in the laboratory by using stereo microscopy (16MZ, Leica). Further morphological details of biofilms and microorganisms (*Fungi*,* Bacteria*,* Archaea*) were detected by light, fluorescence (Axiovert 100, Zeiss), and scanning electron (JSM 630F, Jeol Freising, Germany) microscopy. Prior to light and fluorescence microscope observations, the in situ cell labeling of biofilms and fungal hyphae was performed using modified safranin staining (Hoque et al., 2007) and DAPI (4ʹ,6‐diamidino‐2‐phenylindole) staining.

For scanning electron microscope (SEM) observations, samples (biofilms, fungi) from PBS buffer (pH 7.4) suspensions were fixed at first with 1% glutaric aldehyde for 15 min and then with 2% osmium tetroxide in PBS buffer (pH 7.4). The samples were dehydrated in increasing ethanol gradients (see above). Then, uncut samples were sprayed with nanogold particles prior to observations with scanning electron microscopy at 5–15 kV in the secondary electron mode. The EDX elemental analysis of biofilms and EH11 cells was carried out using an eXL EDX system (Oxford Instruments, High Wycombe, UK). For this purpose, X‐ray quanta were detected by a silicium detector and analyzed by an eXL EDX system using Link analytical software (Hoque & Fritscher, 2016).

### 2.7 Adsorption and accumulation of metals by fungi

The metal accumulation screening assays were conducted in falcon tubes (50 ml) as described (Hoque & Fritscher, 2016). The biological materials (biofilms, Künzing spring; fungal spores, EH11) were treated with defined concentrations (100 μg/L, 1000 μg/L, 10000 μg/L, 50000 μg/L) of single or mixed ionic metals (Cr, Cd, Co, Cu, Al, Au, Ag, Ni, Ti, U, Pb, Zn) for 48 h. The samples were incubated at ambient temperature (25°C) under shaking (100–120 rpm). After incubation, the samples were centrifuged for 3 min at 4000g in order to separate the biological materials from the water. Water was analyzed for residual concentration of metal ions by ion chromatography (Dionex 500 system, see above). For the analysis of arsenics, the samples were treated with HCl (1:1 v/w) and cysteine (1:1 v/w) for 24 h.

The system was calibrated by using 0, 1, 10, 100, 500, and 1000 μg/L concentrations of respective metal standards, whereby the calibration curves with 95% confidence intervals were calculated by linear regression analysis (r^2 ^= 0.99). The standard deviations of measurements of each data point (*n* = 3) were maximum ± 5%.

### 2.8 Ferrihydrite reduction

Spores with mycelia were collected from 3‐ to 4‐week‐old malt extract‐agar culture plates of anoxically grown [80% CO_2_‐20% N_2_ (v/v) atmosphere] *Mucor hiemalis* EH11 and *Penicellium chrysogenum* EH31 under anaerobic conditions. Each time ~5 × 10^6^ spores (ca. 1 cm^2^ mycelium) were incubated in triplicates in an optimized liquid culture medium containing selected electron donor (e.g., acetate, 50 μmol L^−1^) and electron acceptor ferrihydrite [Fe(III), 10 mmol L^−1^] under anoxic [80% CO_2_–20% N_2_ (v/v)] conditions (see above). The reduction of ferrihydrite into Fe(II) as a parameter of growth and e‐acceptor utilization was rapidly determined at 578 nm in well plates (Victor^3^ Multilabel Plate Reader, Model 1420, Perkin Elmer) using ferrozine solution (Stookey, 1970).

### 2.9 Statistical analysis

The statistical analysis was performed using nonparametric Mann–Whitney *U*‐tests according to Weber (1986).

### 2.10 Strain deposit

The pure culture of aquatic *M. hiemalis* strain EH11 was deposited at the DSMZ (Braunschweig, Germany) under the accession number DSM 16292.

## 3 RESULTS

While exploring occurrence of biofilm and fungi in subsurface sediment samples and in sulfidic spring waters with low oxygen concentrations, we succeeded in discovering an anoxic biofilm along with a unique *Mucor hiemalis* strain EH11 from a methane‐sulfidic salty spring named Künzing spring (Bavaria, Germany). In general, this spring belongs to methane–sulfide–sodium chloride–hydrogen carbonate spring type (spring type 4). It served as a healing thermal spring for the Romans since first century A.D. and was famous in the northern region of the Alps. The history of Künzing dates back 7,000 years.

### 3.1 Site description and hydrogeochemistry of Künzing spring

Bavarian groundwater landscapes are divided into 29 groundwater regions; some of them around Künzing (EH11 site) were previously visualized in a graphic (Hoque & Fritscher, 2016). Künzing spring is located at the border of groundwater region “Crystalline Rocky Bavarian Forest” near Czech Republic and the “Tertiary Hilly Land”. The flavoglacial marble rock region is located along the rivers Danube and Isar. The crystalline Rocky Mountains soaring in the north‐eastern part of Bavarian Forest have a border with Danube disturbance zone at the molassic bed and its mesozoic plate. In the swampy Danube valley between Künzing and Pleiting, 4‐m‐thick layers of a bore hole were displaced deeper 30 m below, wherefrom strong odorous methane‐enriched sulfidic water flows at a rate of 3.5 L/s. At this disturbed water‐conducting channel, some pore water from buried marine sources of the earth's crust accumulates and rises up quickly where it is enriched with methane while passing through warm geological upper layers, and thus, spring water becomes mild hydrothermal (mean temperature 19.1°C) and has a salty marine‐like character (Na^+^: 164.7–398.5 mg L^−1^) with no detectable DOC. Bubbling methane and highly reducing (E_h_: −241 to −253 mV) conditions keep Künzing spring's water and its biofilms in an anoxic state ((≤ 0.1 mg O_2_ L^−1^, Table 1). According to tritium measurements, the water of this spring is also the oldest deep ground water among sulfidic springs and is located near all presently known thermal springs of this region.

**Table 1:** Chemical and physical data of methane‐sulfidic Künzing spring water.^a^

Parameters/Springs	Künzing	Teugn^d^
Spring discharge^b^	210 L min^−1^	150–200 L min^−1^
**Temperature**	**18.9–19.2°C**	12.4–12.7°C
**Electrical conductivity**	**1310–1320 μS cm^−1^**	665–697 μS cm^−1^
pH	7.3–7.5	4.7–6.4
**Redox potential (E_h_)**	**−241 to–253 mV**	−192 to −215 mV
**Oxygen (mean)**	**≤0.1 mg L^−1^**	0.7–1.2 mg L^−1^
H_2_S	< 1.0 mg L^−1^	n. m.
Cations and total metals:		
**Na^+^**	**164.7–398.5 mg L^−1^**	50.7–54.7 mg L^−1^
K^+^	10.1–10.5 mg L^−1^	6.5–6.9 mg L^−1^
NH_4_^+^	n. d.	n. d.
Mg^2+^	8.3–9.4 mg L^−1^	22.7–22.9 mg L^−1^
Ca^2+^	12.6–47.3 mg L^−1^	72.7–74.2 mg L^−1^
Mn^2+^	2.5–2.7 mg L^−1^	7.6–9.5 mg L^−1^
Ba^2+^	25.8–45.5 μg L^−1^	47.0–58.2 μg L^−1^
Total Co	< 1 μg L^−1^	0–16.0 μg L^−1^
Total Cu	n.d.–14.4 μg L^−1^	1.1–7.6 μg L^−1^
Total Fe	15.6–20.2 μg L^−1^	9.8–16.7 μg L^−1^
Li^+^	202.0–279.4 μg L^−1^	41.8–49.5 μg L^−1^
Sr^2+^	286.1–1005.4 μg L^−1^	78.9–117.2 μg L^−1^
**Zn^2+^**	**n. d.**	265.2–288.4 μg L^−1^
Anions:		
**Total S^2−^**	**0.7–0.9 mg L^−1^**	1.1–1.3 mg L^−1^
**Cl^−^**	**320.1–354.5 mg L^−1^**	25.5–26.3 mg L^−1^
NO_3_^−^	n. d.	0.2 mg L^−1^
SO_4_^2−^	n. d.	16.1–26.2 mg L^−1^
HCO_3_^−^	570.0 – 574.0 mg L^−1^	420.6–420.9 mg L^−1^
**DOC** c	**n. d.**	0.4–1.1 mg L^−1^
**CH_4_ (emission)** e	**7 m^3^ d^−1^**	n. d.

a The chemical and physical data of the Künzing spring as compared to control spring Teugn are given (September 2002). Some statistically significant (Mann‐Whitney test, α=0.05) important chemical data of Künzing spring are shown in bold font.

b Varies depending on metereological parameters

c DOC: Dissolved Organic Carbon, n. d., not detectable, n.m., not measured

d Hoque et al. (2007)

e Carle (1975)

### 3.2 Biofilm of Künzing spring and its biodiversity

**Figure 1:**
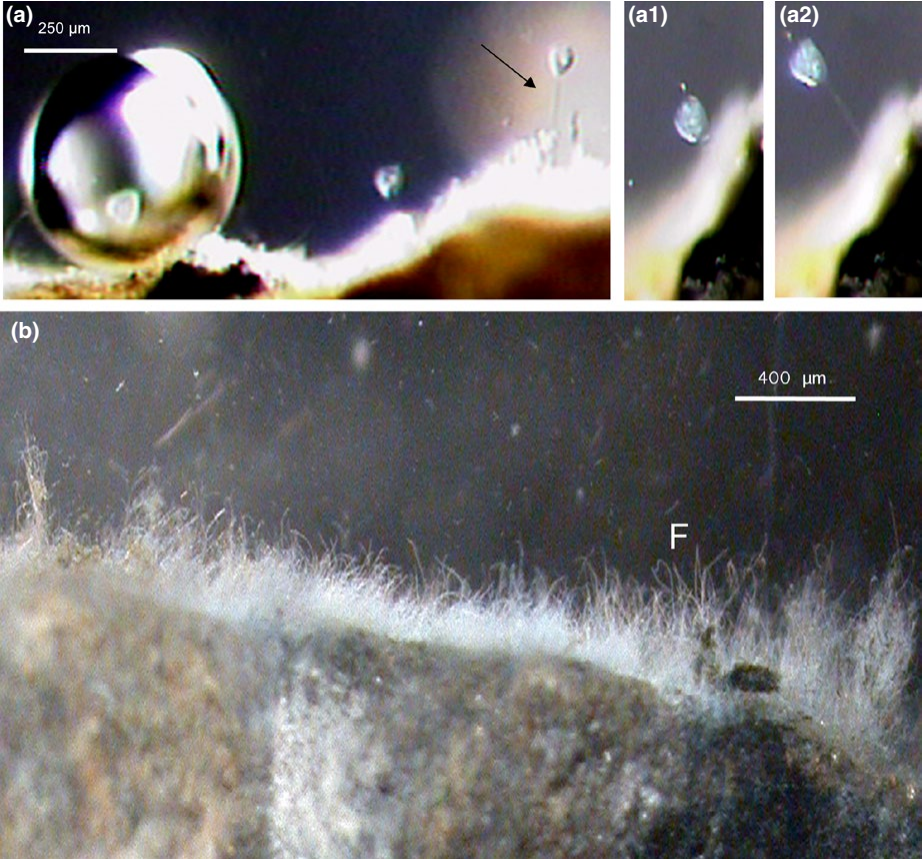
Ecological environment of ciliates and EH11 under low‐oxygen methane‐bubbling conditions of the sulfidic Künzing spring, (a) Sessile ciliate *Vorticella microstoma* near a methane bubble, (a) Springing of ciliate during rise of gas bubbles, (a1) Return of ciliate after the rise of gas bubbles above the ciliate, (a2) Springing up again, (b) Sessile EH11 (F) on a rock in Künzing spring

The ecological environment of ciliates and fungal hyphae (EH11) under low‐oxygen methane‐bubbling conditions of the sulfidic salty spring Künzing is shown in Figure 1. A sessile ciliate *Vorticella microstoma* springing up during the rise of a methane gas bubble and springing back after the bubble had risen above the ciliate as well as sessile thread‐like fungal hyphae (EH11, F) were observed under in situ intact conditions by 3D microscopy on a rock surface. Due to continuous bubbling of methane, the biofilms of Künzing were compelled to fix themselves to rocks (Figure 1).

To test the hypothesis that microbial community members may live also strictly anaerobically in water‐saturated groundwater systems, we developed culture‐dependent and culture‐independent methods for systematic investigations of fungi under anoxic groundwater–sediment systems. Culture‐dependent methods included isolation, cultivation, morphological comparison, and mating behavior. In contrast, culture‐independent methods, for example, phase contrast microscope and SEM investigations as well as molecular biological procedures (FISH; PCR, cloning, sequencing, and phylogenetic analysis) were modified and applied for aquatic anaerobic fungal research (Hoque & Fritscher, 2016; Hoque et al., 2007).

**Figure 2:**
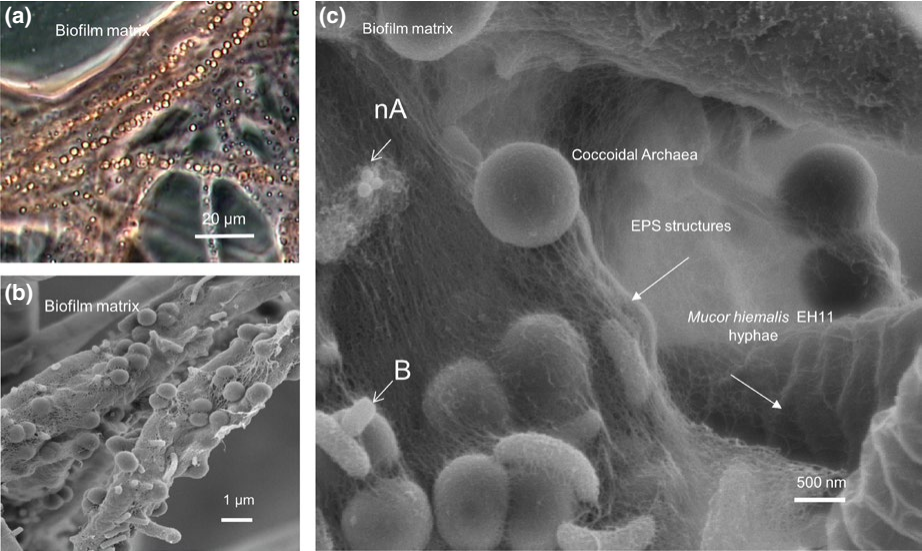
Components of methane‐sulfidic spring biofilm of Künzing, (a) Phase contrast light microscopy of biofilms with coccoidal *Archaea* arranged like a Chain of Pearls, (b) Scanning electron microscopy of biofilm showing dense bed of coccoidal *Archaea*, (c) Scanning electron microscopy of biofilm at a higher magnification showing micro‐ and nano (nA)‐coccoidal *Archaea*,* Bacteria* (B), EPS structures, and fungus EH11

In the biofilm matrix of the sulfidic Künzing spring, phase contrast light microscopy revealed coccoidal *Archaea* arranged like a Chain of Pearls. More details were observed by SEM, as, for example, coccoidal *Archaea* with EPS structures, *Bacteria* and side‐by‐side occurring typical fungal hyphae EH11 (Figure 2).

**Figure 3:**
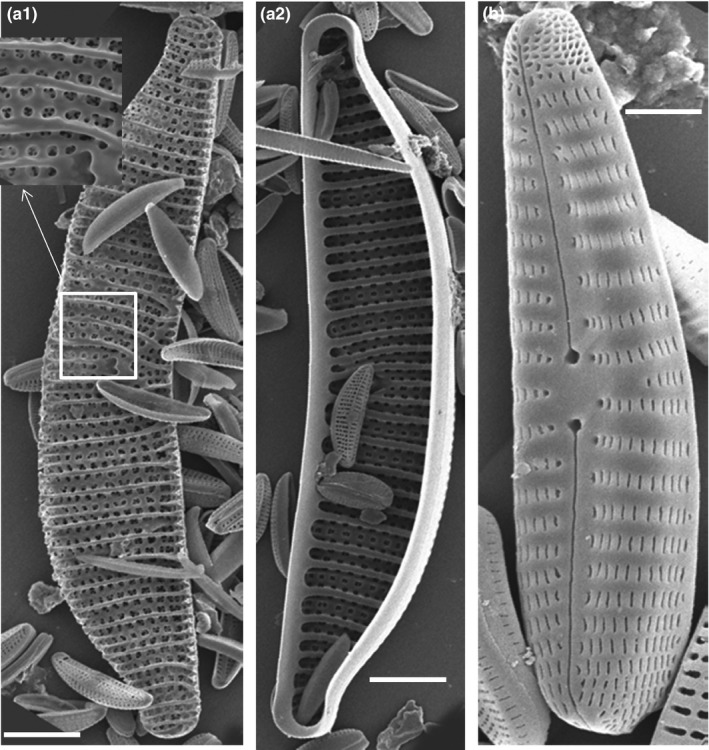
SEM of two typical diatoms along with smaller diatoms from Künzing spring biofilm. *Epithemia adnata*, (a1)‐ outer shell with exploded view of a portion, (a2)‐ inner shell; b. *Rhoicospenia abbriviata*. White bar indicates 5 μm

As diatoms are mostly good indicators of a spring's water quality, we investigated diatoms of the sulfidic springs and found eight different diatom groups. In the Künzing spring, we could detect mostly anaero‐ and halotolerant diatoms representing about 30% of all the diatom species found in the sulfidic springs of Bavaria (Germany). SEM analysis showed the occurrence of numerous anaero‐ and halotolerant diatoms, which are typical for Künzing's nearly anoxic marine environment. Some of the identified anaero‐ and halotolerant diatoms include, for example, *Achnanthes lanceolata, Achnanthes langenburgeana, Achnanthes silhaver*,* Amphora pediculus*,* Diadesmis gallica* ssp. *perpusilla*,* Epithemia adnata*,* Fragilaria laptostauron* var. *martyi*,* Fragilaria ulna*,* Navicula minutissima, Navicula papulacea, Navicula tripunctata, Rhoicosphenia abbreviata*,* Stauroneis smithii* (Figure 3, S1).

### 3.3 18S and ITS cloning and identification of *Mucor hiemalis* EH11

Figure S2 shows 18S‐ and ITS‐primer locations on a schematic representation of 18S, ITS‐1, 5.8S, ITS‐2, and LSU rRNA regions. In addition to the 18S cloning and sequencing technique (Hoque et al., 2007), we developed an alternative ITS cloning and sequencing method (Hoque & Fritscher, 2016). The PCR products after amplification of Künzing spring biofilm's DNA using the best primer pair, ITS1F (f) and ITS(b) (Figure S2, see Materials and Methods), were cloned in *E. coli* p‐GEMT vector system. Maximum 56 clones per clone library were picked, and plasmid DNAs were isolated. After cycle sequencing of plasmid DNA templates, ITS sequences obtained were assembled. Sequence comparison successfully showed the presence of *Mucor hiemalis* as a crucial fungus in 67% of the investigated sulfidic springs. This technique is now ready for use to explore the occurrence of fungi in incubation media as well as in groundwater–sediment systems.

Amplification of the 18S rRNA gene sequence by using selective *Mucor* primers and the phylogenetic tree construction of aquatic *Mucor hiemalis* based on 18S rRNA sequence data showed unequivocally the identity of Künzing's *Mucor* species as *Mucor hiemalis* f. *hiemalis* (plus strand).

Fluorescence in situ hybridization (FISH) analysis of Künzing's biofilm showed that the aquatic fungus *M. hiemalis* EH11 is represented in biofilms by 10%, *Bacteria* 60% (α*‐Proteobacteria* 20%, β*‐Proteobacteria* 5%, γ*‐Proteobacteria* 10%, δ*‐Proteobacteria* 5%, *Cytophagas‐Flavobacteria* 10%, *Bacteria* with high G + C concentrations 10%), and *Archaea* 30%. In the methane gas–sulfide–salt‐spring types, δ*‐Proteobacteria* were detected up to 5% in the mild hydrothermal springs and up to 10% in the iodine‐containing methane gas‐sulfidic salt springs. The highest number of *Archaea* was found in the methane‐sulfidic salt springs. In this spring type, 20% *Euryarchaeota* were detected by FISH analysis using the EURY498 probe. *Crenarcheota* (10%) were found only in the mild hydrothermal methane‐sulfidic salt spring of Künzing using the CREN499 probe (Hoque & Fritscher, 2016; Hoque et al., 2007).

### 3.4 Adaptation and spiral morphology of filamentous microorganisms in methane‐sulfidic spring of Künzing

The ecological environment of ciliates and EH11 under low‐oxygen methane‐bubbling conditions of the sulfidic Künzing spring could be described by a sessile ciliate *Vorticella microstoma* that springs up during the rise of a gas bubble and returns after the gas bubble rises above the ciliate.

**Figure 4:**
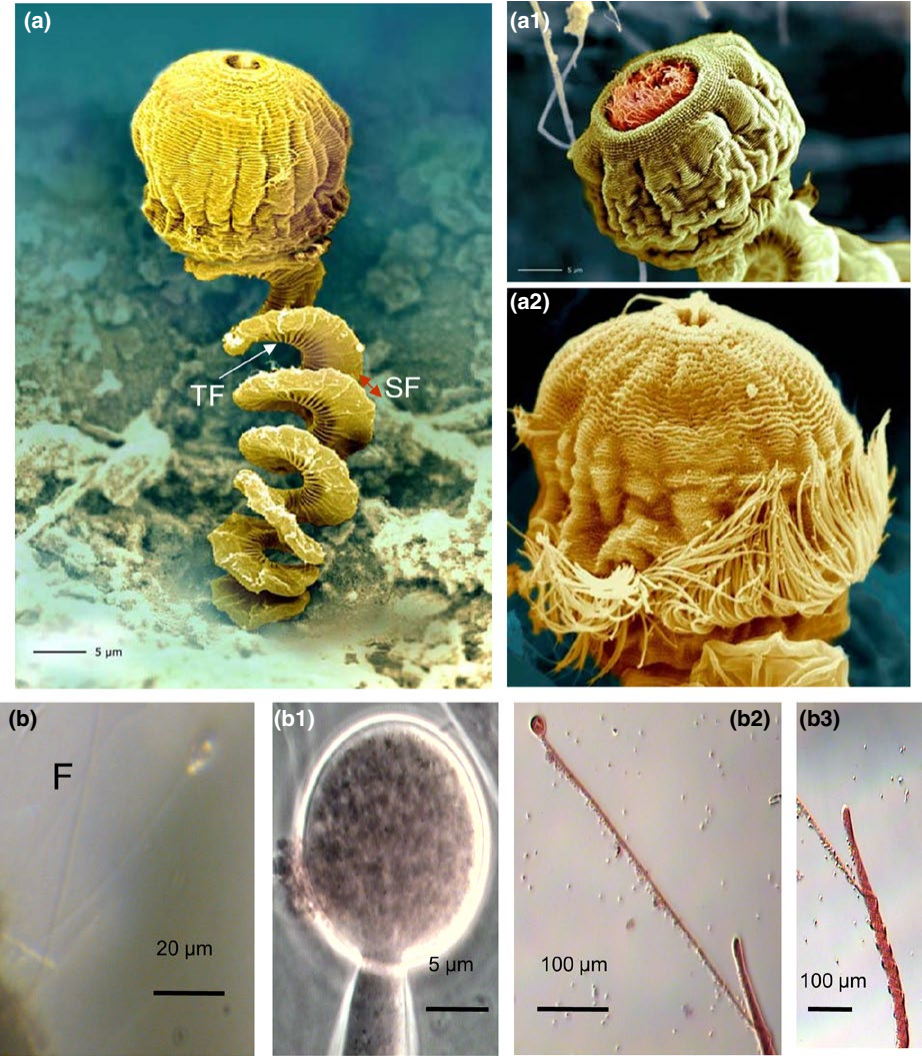
Detailed morphology of ciliate and fungus grown under low‐oxygen methane‐bubbling conditions of the spring Künzing, (a) SEM image (pseudocolor imaging) of a sessile ciliate *Vorticella microstoma* showing spring‐like stalk. Note fine structures of the spring‐like stalk (spasmoneme) revealing transverse fibrils and regular transverse outer stabilizing fibers (SF); (a1) An older *Vorticella* with opened peristome and filamentous capture device (red), (a2) Younger *Vorticella* with a hairy ornamental structure, and (b) Sessile EH11 (F) on a rock, (b1) sporangium with a sporangiophore, (b2) spiral nature of EH11's second sporangiophore around the primary sporangiophore, (b3) exploded view of b2

The morphology of ciliate and fungi from low‐oxygen methane‐bubbling conditions at the Künzing spring showed some peculiarities, SEM imaging (pseudocolor imaging) of sessile ciliate *Vorticella microstoma* was used to visualize structural details of its spring‐like stalk. Fine structures of the spring‐like stalk (spasmoneme) revealed transverse fibrils (TF) and regular transverse outer stabilizing fibers (SF) (Figure 4a). An older *Vorticella* could be observed with an opened peristome and a filamentous capture device (red, Figure 4a1), whereas a younger *Vorticella* with a hairy ornamental structure showed a closed peristome (Figure 4a2).

Sessile EH11 (Figure 4b) on rock with typical sporangiophore and sporangium are shown after modified safranin staining. Similar to the spiral morphology of the sessile ciliate *Vorticella microstoma*, the morphology of the fungus EH11 from the methane‐sulfidic salt spring featured a sporangium with a sporangiophore (Figure 4b1) which formed an additional second carrier sprorangiophore for the spiral winding of the actual main sporangiophore (Figure 4b2 and b3). This adaptation was detected only in situ, it was not possible to cultivate this phenotype for a long time. The spiral forms of adaptation are common in this spring. The in situ occurrence of this fungus in biofilms with other microorganisms together was documented by SEM (Figure 2).

Culture‐dependent methods included cultivation on anaerobic fungal solid and in liquid media as well as testing of substrates by addition of glucose, cellobiose, acetate as an electron donor and nitrate, sulfate, ferrihydrite/AQDS as an electron acceptor. Using such a method we isolated and identified various strains of *Mucor hiemalis* in different sulfidic spring biofilms. From the sessile biofilm (Figure 1), EH11 was isolated, purified by repeated cultivations on malt extract agar solid medium, characterized by strain antagonism tests and identified as a plus strand of *Mucor hiemalis* f. *hiemalis* by matching with the corresponding minus strand as previously described (Hoque et al., 2007).

### 3.5 Effects of salt on *Mucor hiemalis* strain EH11

The Künzing spring near Passau (Bavaria) shows the highest salt concentration among all the investigated sulfidic spring waters because this spring is fed by a marine molassic bed (Table 1). The fungus EH11 was challenged with sodium chloride concentrations between 5 and 25 mg/L for 48 h. EH11 tolerated these salt concentrations and grew normally without utilizing salt in the water.

### 3.6 Accumulation of ionic metals by Künzing's biofilm

In contrast to Marching spring's biofilm with the ability of mercury and zinc removal (Hoque & Fritscher, 2016), the Künzing spring's biofilm possessed no ability to accumulate mercury, chromium, and zinc, but it accumulated some other metal ions to similar degrees. Metal‐ion accumulation investigations were carried out also to characterize and assess the biofilm's ability for metal detoxification.

The in situ enrichment of various metals by the biofilms was up to 8.2 × 10^5^‐fold (Al 8.2 × 10^5^‐fold, Cd 75 × , Co 350 × , Cr 0 × , Cu 970 × , Fe 1.1 × 10^5^‐fold, Li 2.2 × 10^4^‐fold, Mn 6.500 × , Ni 1.400 × , Pb 3.900 × , Sr 19 × , Zn 0 × , Hg 0 × , As 0 × ).

### 3.7 Search for other anaerobic fungi in contaminated ground water sediments for comparison

We isolated two fungi from 10‐ to 14‐m‐depth ground water sediments of a Quaternary sandy aquifer at a former Gasworks (Düsseldorf‐Flingern, Germany) by using hollow‐stem auger drilling (Anneser et al., 2010). These fungi utilized glucose and cellobiose as an e‐donor, and ferrihydrite/AQDS as an e‐acceptor under strict anaerobic conditions. When grown aerobically on solid medium, a consortium of at least two fungi/strains from anaerobic liquid media incubated with cellobiose as an electron donor and ferrihydrite/AQDS as an electron acceptor was shown to exist and were observed by stereomicroscopy. One of these fungi was identified morphologically as *Penicillium chrysogenum* strain EH31, and used for comparison with EH11 (see below).

The optimum temperature of growth of aquatic fungi under anoxic conditions was detected within ambient temperature (20–30°C). As, for example, aquatic *Mucor hiemalis* mycelium grew optimally 10 mm day^−1^ at this temperature range, but it even grew 1 mm day^−1^ near freezing temperature (0.3°C).

### 3.8 Morphological system responses of EH11 under strictly anaerobic conditions

*Mucor hiemalis* EH11 from methane‐sulfidic spring water grew under various anaerobic conditions using various e‐donors (cellobiose, acetate) and e‐acceptors (nitrate, sulfate, ferrihydrite; AQDS as e‐shuttle).

**Figure 5:**
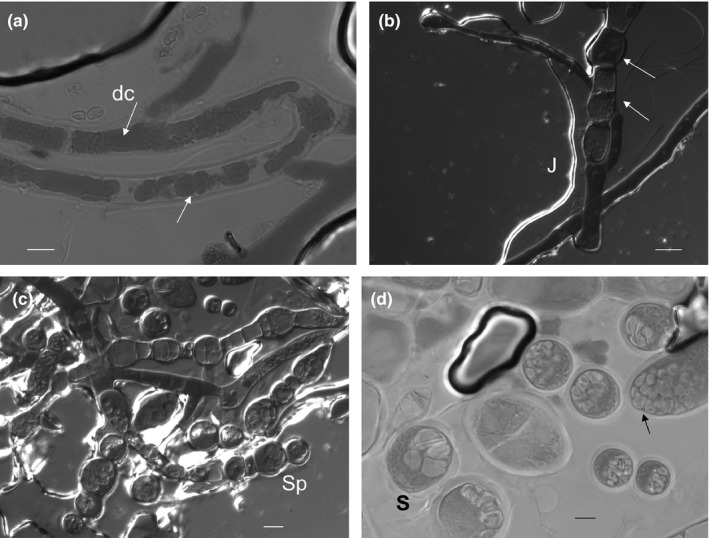
Variation in morphology of *M. hiemalis* EH11 under strict anaerobic culture conditions (e‐donor: cellobiose, e‐acceptor: nitrate). (a) Formation of dense material (dc, arrows) in nonseptate hyphae, (b) initialization of septate formation, cell division, cell swelling, thickening of intracellular materials in sporangium‐like formations of EH11 (arrows near a jute fiber, J) under strict anaerobic conditions imaged by transmission differential contrast microscopy, (c) strong cell division, cell swelling, initialization of pseudosporangiospores, and detachment of intracellular pseudosporangium (Sp) instead of terminal sporangium release as observed by differential contrast microscopy, (d). Release and ripening of detached cells (S, pseudosporangium) as well as intracellular division into numerous pseudosporangiospores within the pseudosporangia. Scale bar indicates 5 μm

Under strict anaerobiosis, peculiar micromorphological changes in EH11 were observed. Microscope investigations on EH11 under anaerobic conditions, for example, using cellobiose as an e‐donor and nitrate as an e‐acceptor, revealed at first initial formation of highly dense cellular (dc) materials in zygomycete‐typical nonseptate hyphae (Figure 5a). In continuation of anaerobiosis, occurrence of unusual septate formations in EH11's hyphae in otherwise nonseptate hyphae dividing the hyphae into smaller cell compartments was detected (Figure 5b). Enhanced cell division and accumulation of sporangiospore‐like formations, denoted as pseudosporangiospores, in these dividing cells, termed pseudosporangium, were observed (Figure 5b–c). Stimulated release of pseudosporangium and ripening of numerous pseudosporangiospores in pseudosporangium of EH11 under strict anaerobic conditions is a peculiarity worth mentioning (Figure 5d), which differed from chlamydospore formations of *Mucor* species. Thus, strong cell divisions to generate precursors of intracellular pseudosporangium instead of terminal sporangium are visible in EH11 under strict anaerobiosis.

**Figure 6:**
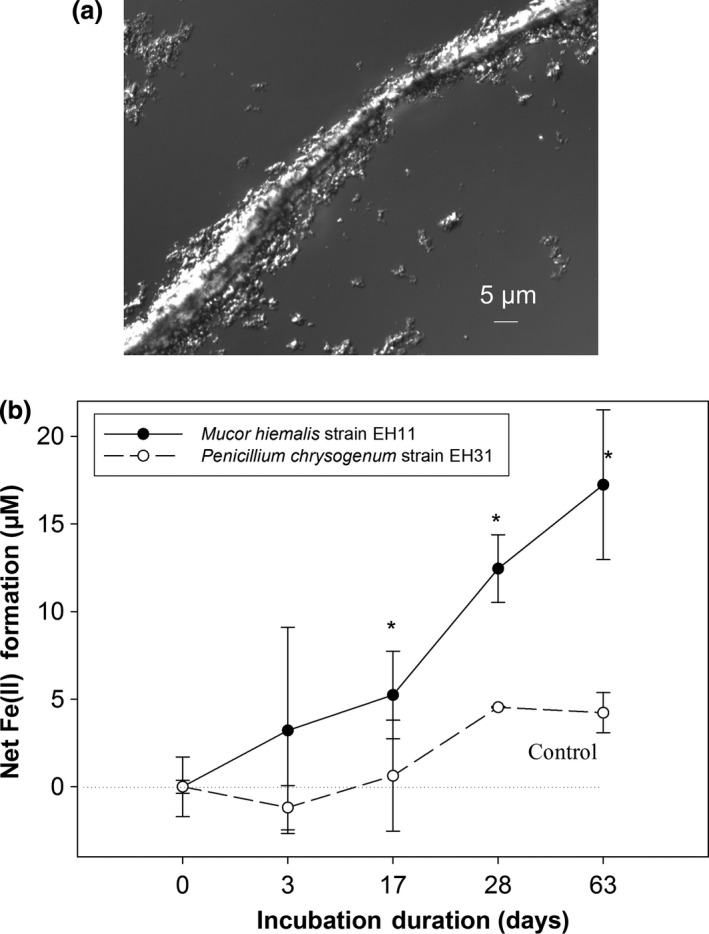
Accumulation of ferrihydrite [Fe(III) species] and its reduction by EH11 under strict anaerobic conditions. (a) Fungal filament with ferrihydrite around the hypha grown in an anaerobic liquid medium with cellobiose as an electron donor and ferrihydrite as an electron acceptor with AQDS as an electron shuttle, (b). Ferrihydrite reduction [Fe(II) formation] by EH11 (solid line) compared to control *Penicillium chrysogenum* EH31 (isolated from anaerobic sediments) in an anaerobic liquid medium with acetate as an e‐donor and ferrihydrite as an e‐acceptor depending on incubation duration (days). The statistical significance of data points is annotated by * symbol (Mann–Whitney test, α = 0.05)

When EH11 was grown in anaerobic liquid medium with cellobiose as an electron donor and ferrihydrite as an electron acceptor along with AQDS as an electron shuttle, ferrihydrite was selectively accumulated by EH11 at the hyphal walls, visible as a strong sheathing of fungal filament with ferrihydrite around the hypha, as if EH11 tried to harness electrons from the e‐donor ferrihydrite for energy production (Figure 6a, top).

Incubation of *Mucor hiemalis* strain EH11 in a strictly anaerobic medium containing acetate (50 mmol L^−1^) as an electron donor and ferrihydrite (10 mmol L^−1^) as an electron acceptor with AQDS (10 mmol L^−1^) as an electron shuttle showed that the strain EH11 has the ability to grow chemoautotrophically, without fermentation similar to chemoautotrophic *Bacteria* under anoxic conditions (see below).

### 3.9 Removal of toxic metals by EH11

Aquatic strains of *M. hiemails* (EH4–EH12) responded differently to various heavy metal concentrations. The strain EH11 fixed more than 80% of the applied ionic metals (Al 98.9%, Cd 80.3%, Co 80.6%, Cr 89.5%, Cu 87.2%, Ni 82.1%, Pb 97.1%, Zn 83.6%) on the spore surfaces (Table 2). Mercury was accumulated only by EH8 of the sulfidic Marching spring (Hoque & Fritscher, 2016), but arsenic was not accumulated by any of the tested fungal strains.

**Table 2:** Biosorptions of ionic metals and metalloids by Künzing's biofilms and EH11*

Metal/Spring	Al Bf F	Cd Bf F	Co Bf F	Cr Bf F	Cu Bf F	Ni Bf F	Pb Bf F	Zn Bf F
Teugn	21.5 0	96.9 22	98.5 28.5	48.8 31.7	94.1 44.6	94.3 0	98.1 49.4	51.7 50.5
Künzing	90.7 98.9	96.6 80.3	99.5 80.6	92.4 89.5	94.8 87.2	99.7 82.1	96.6 97.1	86.4 83.6

The matched values in biofilms and fungus from selected springs are shown in bold font. The standard deviations of measurements (*n* = 3) are maximal 5%.

*The mean percentages of ionic metal removal from water by bioabsorption are given for each heavy metal cation applied (1,000 μg/L) in a mixture to biofilms (Bf) and corresponding fungal cultures (F: EH11) of Künzing spring in comparison to control spring Teugn and its fungus *Mucor hiemalis* EH7.

The efficiency of metal accumulation by EH11, except for mercury, was similar to that of the strain EH8, which was isolated from a methane‐sulfidic salt spring with similar chemical compositions (Hoque & Fritscher, 2016), but the mean temperature of the Künzing spring is about 8.6°C higher than the other spring.

### 3.10 Utilization of acetate as an electron donor and ferrihydrite as an electron acceptor with AQDS as an electron shuttle by a new strain EH11 of the fungus *Mucor hiemalis*

The ferrihydrite reduction [Fe(II) formation] by EH11 (solid line) compared to control *Penicillium chrysogenum* EH31 (isolated from anaerobic sediments) depending on incubation duration under strict anaerobic conditions is demonstrated in Figure 6 (below). Using acetate (50 mmol L^−1^) as an electron donor and ferrihydrite (10 mmol L^−1^) as an electron acceptor with AQDS (10 mmol L^−1^) as an electron shuttle, it can be shown that the strain EH11 continuously reduced Fe(III) (ferrihydrite) into Fe(II) from the ouset, but after an initial lag period of about 17 days statistically significantly stronger than *P. chrysogenum* EH31 (Mann–Whitney test, α = 0.05).

These special microbial habitat conditions promoted the selective growth of salt‐tolerant micro‐ and nanosized *Archaea*, mainly *Euryarchaeota* and *Crenarchaeota*, diverse *Bacteria* and aquatic anaerobic fungus EH11 as shown by FISH analysis (Hoque & Fritscher, 2016; Hoque et al., 2007), and numerous diatoms in the rock‐sessile biofilm. Culture‐dependent and ‐independent methods revealed the biofilm's microbial composition and led to characterization and/or identification of the key players of the biofilm's functions in Künzing spring water.

EH11 and other microorganisms can be suggested to be immigrants to the Künzing spring's biofilm, reaching it by any means of dispersal (wind, surface, and deep marine water), but adapting themselves over years to methane and mild hydrothermal‐sulfidic salty marine‐like habitat conditions.

## 4 DISCUSSION

Investigations on Künzing spring's biofilm and its community members revealed their special biodiversity, ecological habitat, and adaptation under bubbling methane. Mild hydrothermal, methanogenic, sulfidic, anoxic, and the salt‐rich marine ecology of Künzing spring enabled the formation of a special sessile biofilm consisting of diverse *Archaea* (*Euryarchaeota* and *Crenarchaeota), Bacteria*,* Protozoa*, fungus, and diatoms (Figure 1, 2). Abundant *Archaea* were connected with each other by EPS structures to form Chains of Pearls in association with fungal hyphae (Figure 2). Similarly, a fungal‐prokaryotic consortium was detected in the subseafloor igneous crust at the basal–zeolite interface (Ivarsson et al., 2015). Bengtson et al. (2014) also described subseafloor microbial communities and Archaeal Chains of Pearls in association with fungal hyphae as well. It is surprising that similar morphology and community structure were found in both environments, both extreme and geophysically similar, but geographically so far apart. The sessile fungal hyphae with the Archaeal EPS structures built a 3D matrix, where *Bacteria* and diatoms were embedded. Diatoms occurring in the Künzing spring's biofilm were halophilic and anaero‐tolerant and reflected the water quality of this microbial habitat indicating sulfidic marine‐like salty ecology of Künzing spring, where only special *Protozoa* and fungi can grow. The protozoan ciliate *V. microstoma* and the fungus EH11 were found there. The nearly anoxic igneous oceanic crust circulated by seawater and highly saline hydrothermal fluids was described as the earth's largest fungal habitat (Ivarsson, Bengtson, & Neubeck, 2016). A search of fungal diversity in deep‐sea hydrothermal ecosystems also revealed the occurrence of fungi, many of them were unknown and some of them were from higher taxonomic levels like *Chytridiomycota*,* Ascomycota,* and *Basidiomycota* (Calvez, Burgaud, Mahe, Barbier, & Vandenkoornhuyse, 2009). Similarly, the *Zygomycete* EH11 was isolated from a nearly anoxic sulfidic salt spring fed by marine mollasic bed water, which is comparable to marine habitats of numerous fungi described by Ivarsson et al. (2016). Thus, strictly anaerobic fungi may occur in anoxic deep groundwater–sediment systems. The feasibility of finding many other anaerobic fungi in anoxic‐reducing aquifers is high. Occurrence of some other fungi in partially anoxic spring waters was also detected, whereby presence of chytrid‐like fungi in a partially anoxic spring water environment was observed by us (Fritscher, 2004).

Microbial adaptation to Künzing spring's extreme aquatic environment was only made possible by selective specialists mutually benefitting from complementary microbial community members of the special biofilm. Results demonstrate that microbial life in the aquatic ecosystem of Künzing spring adapts itself to the harsh anoxic methane‐sulfidic salt conditions. Continuous bubbling of methane at a rate of 4.86 L min^−1^ (recalculated) and highly reducing conditions in relatively warm (mean temperature: 19.1°C) methane‐sulfidic salt spring water of Künzing lowered the oxygen level ≤ 0.1 mg L^−1^ (Table 1), similar to near anoxic deep marine–like habitat conditions of diverse microorganisms. The spiral spring‐like structures of the ciliate *V. microstoma* and the fungus EH11 provide evidence of special adaptation to cope with the pressure created by bubbling methane. Both can spring up with the rising methane bubble and come back again after the bubble escapes the biofilm. EH11 fixes the biofilm to the rock so that a sessile biofilm with fungal hyphae and *Archaeal‐Bacterial* EPS structures is formed that can hold and protect the rest of the microorganisms.

The identification of EH11 and its successful cultivation in strictly anaerobic liquid cultures (see Materials and Methods) unlocked the door to investigate growth and functions of this crucial member of the Künzing biofilm's microbial community, especially to study adaptive transformations of EH11 under anoxic conditions. It can be suggested that under anaerobiosis, EH11 becomes more productive and competitive, as advocated by Shcherbakova et al. (2010), by switching metabolism (see below) and intensifying asexual reproduction. Strong increment of multiplication ability of fungi during anaerobiosis may also be important for their survival and pathogenic interactions with hosts. Similarly, Tonouchi (2009) reported a fungal *Lecanoromycetes* strain from rice field soil, which developed swollen hyphal cells under anaerobiosis, appearing like conidium and chlamydospore usually not produced under aerobic conditions.

Without fermentation EH11 switched its metabolism completely to anaerobic by utilizing acetate as an e‐donor and Fe(III) as an e‐acceptor: This emphasizes the high adaptation power of anaerobic fungus EH11 similar to iron‐reducing *Bacteria* under adverse anoxic conditions not previously known. Similarly, the reduction of Fe(III) into Fe(II) by anaerobic *Bacteria* was coupled to carbohydrate oxidation (Coates, Councell, Ellis, & Lovley, 1998). Here, we also have evidence that anaerobic fungus EH11 can live like anaerobic *Bacteria* by coupling oxidation of acetate (e‐donor) to reduction of Fe(III) (ferrihydrite, e‐acceptor) into Fe(II) under strictly anoxic conditions. This is the first report about the ecology of a fungus EH11 that is capable of chemoautotrophic *Bacteria*‐like nonfermentive metabolism under strictly anaerobic conditions.

Similar to EH11's hyphal pseudosporangium formation and release, exogenous sporangia formation is regarded as a typical feature of strictly anaerobic polycentric rumen fungi, which could be observed by fluorescence microscopy using 2′‐(4‐hydroxyphenyl)‐5‐(4‐methyl‐1‐piperazinyl)‐2,5′‐bi‐1*H*‐benzimidazole stain (Fliegerova, Hodrova, & Voigt, 2004). Polycentric fungal mycelium shows variations in hyphal forms (tubular and uniform or wide and irregular), sometimes with constricted hyphae at short intervals (bead‐like appearance). Monocentric or polycentric thallus, filamentous or bulbous rhizoids, and mono‐ or polyflagellated zoospores, as well as electron microscope observations of zoospore ultrastructures are often considered as valuable criteria for morphological identification of anaerobic fungi (see above). In contrast to pseudosporangia produced by EH11's hyphal intracellular divisions, chlamydospores from *Mucor circinelloides* hyphae were oval—subglobose shaped without any subdivisions into sporagiospores, but the sporangia of this fungus were subdivided into sporangiospores (Iwen, Sigler, Noel, & Freifeld, 2007). This micromorphological behavior of *Mucor* species under anoxic conditions could be of high importance for the assessment of invasion and pathogenicity of some opportunistic fungi under anoxic–hypoxic conditions. It is worth mentioning in connection with this that some anaerobic fungal zoospores were chemoattracted to phenolic acids (Wubah & Kim, 1996), which could be released during degradations of woody materials.

In general, the fungi are considered as key organisms in ecosystems on the basis of their ecological function and the ability to degrade organic matter (Calvez et al., 2009). Nevertheless aquatic fungus also provides physical matrix to harbor *Archaea*,* Bacteria*, and other microbial community members in biofilms (Hoque et al., 2007). We could show the unusual metal filtration power of EH11 as one of the ecological roles of this fungus in the natural biofilm under nearly anoxic conditions. Practically, EH11 can also be used in combination with other sulfidic spring's *Mucor hiemalis* strains for the simultaneous removal of toxic metal ions from groundwater and industrial waste water with high metal and sulfide concentrations (Fritscher, Hoque, & Stöckl, 2006). Thus, it can be suggested that aquatic anaerobic fungi along with other microbial community members, for example, protozoan *Vorticella microstoma*, play not only a vital role in filtration of toxic ionic metals from water but also in C recycling (Table 2; Shakoori, Rehman, & ul‐Haq, 2004). EH11 is not only ecologically important for the Künzing spring's microbial community concerning physical stability, but also for its functions in elimination of toxic metal ions (Table 2). However, many anaerobic fungi possess enzymes essential for the degradation of ligno‐cellulosic materials for C‐recycling (Chen et al., 2003; Gerbi et al., 1996; Li, Chen, & Ljungdahl, 1997; Yanke et al., 1996). Thus, the ecological role of anaerobic fungi in such an extreme aquatic environment (nearly anoxic sulfidic marine conditions) in the biofilm could be manifold: (1) physical support (Hoque & Fritscher, 2016; Hoque et al., 2007), (2) special enzymes (see above), (3) special chemoautotrophic *Bacteria*‐like metabolism (see results), (4) special asexual reproduction (see results), (5) element recycling (Calvez et al., 2009; Gadd, 2010; Ivarsson et al., 2015, 2016), (6) detoxification of organic toxic substances (Hoque et al., 2007), (7) metal detoxification (Gadd, 2010; Hoque & Fritscher, 2016; Hoque et al., 2007), (8) establishment and exposure of special deep sea‐marine hydrothermal‐like microecosystem to terrestrial surface, and (9) other hitherto unknown beneficial functions. Exploring the current literature on the role of anaerobic fungi in utilization of complex carbohydrates or ligno‐cellulosic materials from plants, we can suggest the role of anaerobic and facultative anaerobic fungus as crucial in the ecosystem. Fungal, Bacterial, and diatom interactions are suggested to modulate the formation of DOC as a carbon‐based energy source of diverse range of microorganisms.

We still know a little about the role, functions, and importance of anaerobic fungi in the earth. The oldest fossil of probable fungi, perhaps anaerobic, found so far dates back to the Proterozoic Eon, about 1.3 Billion years ago (Butterfield, 2005). The similarity of the fungal‐prokaryotic consortium of Künzing spring with that of a deep sea marine subfloor may indicate the hitherto unknown role of the spring's anaerobic fungi in generation, establishment, and exposure of deep sea marine hydrothermal‐like microecosystems for transition of aquatic life to the terrestrial land surface. Further research in future can help unlock the mystery of other interesting fungal ecological functions in strictly anaerobic environments.

## CONFLICT OF INTEREST

No conflict of interest.
