# Supplementary figures and images for: Ecology, adaptation, and function of methane‐sulfidic spring water biofilm microorganisms, including a strain of anaerobic fungus Mucor hiemalis
Source: Microbiologyopen. 2017 May 24;6(4):e00483. doi: 10.1002/mbo3.483 (PMC5552911; doi:10.1002/mbo3.483)

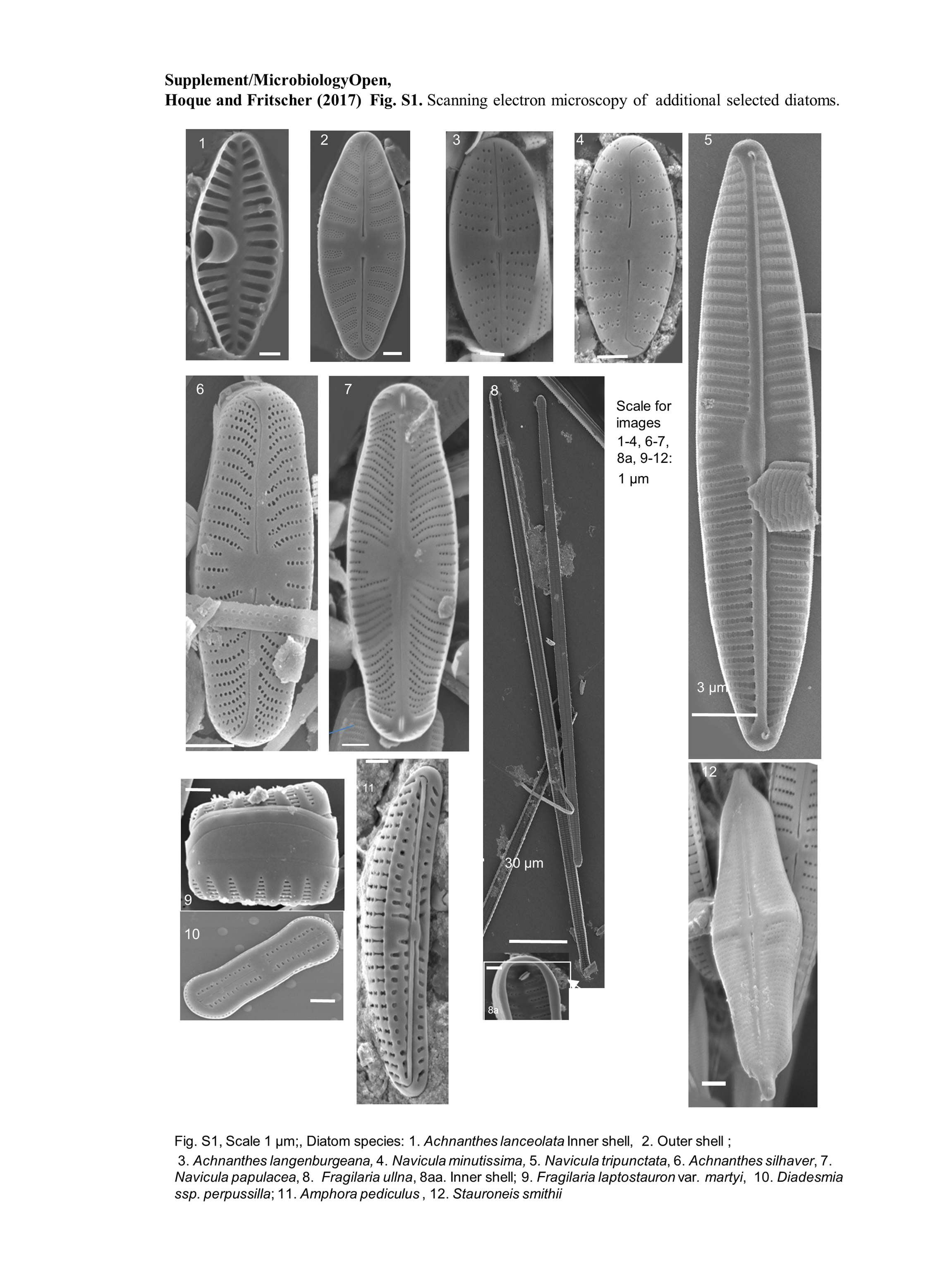

Supplement: Supplementary file 1 [file MBO3-6-na-s001.tif]

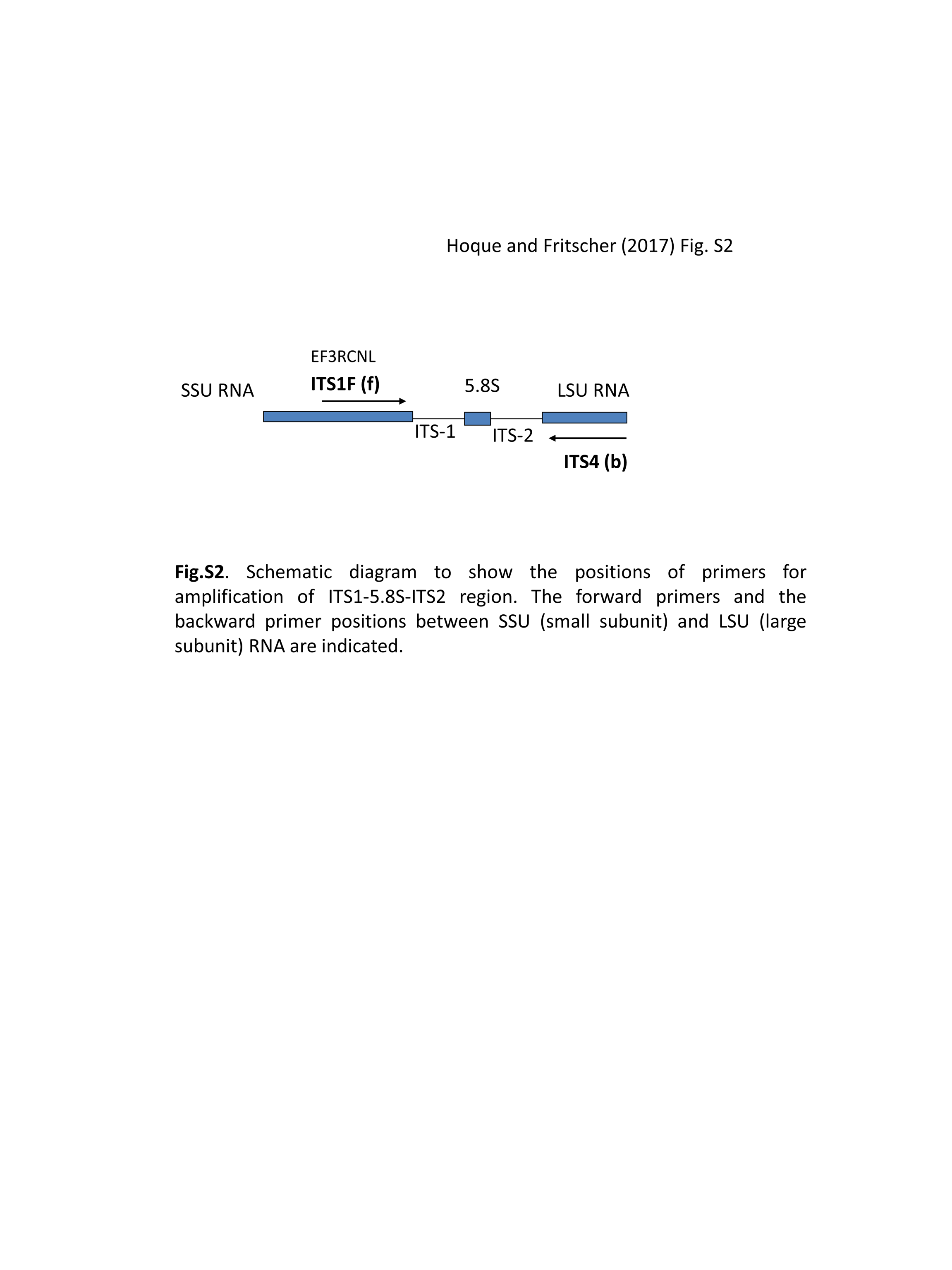

Supplement: Supplementary file 2 [file MBO3-6-na-s002.tif]
